# Prognostic significance of CNSL at diagnosis of childhood B-cell acute lymphoblastic leukemia: A report from the South China Children’s Leukemia Group

**DOI:** 10.3389/fonc.2022.943761

**Published:** 2022-08-10

**Authors:** Lu-Hong Xu, Xu Geng, Ning Liao, Li-Hua Yang, Hui-Rong Mai, Wu-Qing Wan, Li-Bin Huang, Min-Cui Zheng, Chuan Tian, Hui-Qin Chen, Qi-Wen Chen, Xing-Jiang Long, Zi-Jun Zhen, Ri-Yang Liu, Qiao-Ru Li, Bei-Yan Wu, Li-Na Wang, Xian-Ling Kong, Guo-Hua Chen, Jian-Pei Fang, Yang Li

**Affiliations:** ^1^ Department of Pediatric Hematology/Oncology, Children’s Medical Center, Sun Yat-sen Memorial Hospital, Sun Yat-sen University, Guangzhou, China; ^2^ Guangdong Provincial Key Laboratory of Malignant Tumor Epigenetics and Gene Regulation, Sun Yat-sen Memorial Hospital, Sun Yat-sen University, Guangzhou, China; ^3^ Department of Pediatrics, The First Affiliated Hospital of Guangxi Medical University, Nanning, China; ^4^ Department of Pediatric Hematology, Zhujiang Hospital, Southern Medical University, Guangzhou, China; ^5^ Department of Hematology and Oncology, Shenzhen Children’s Hospital, Shenzhen, China; ^6^ Department of Pediatrics, The Second Xiangya Hospital of Central South University, Changsha, China; ^7^ Department of Pediatrics, The First Affiliated Hospital, Sun Yat-sen University, Guangzhou, China; ^8^ Department of Hematology, Hunan Children’s Hospital, Changsha, China; ^9^ Department of Pediatrics, Affiliated Hospital of Guangdong Medical University, Zhanjiang, China; ^10^ Department of Pediatrics, The Third Affiliated Hospital, Sun Yat-sen University, Guangzhou, China; ^11^ Department of Pediatrics, The First Affiliated Hospital of Nanchang University, Nanchang, China; ^12^ Department of Pediatrics, Liuzhou People’s Hospital, Liuzhou, China; ^13^ Department of Pediatric Oncology, Sun Yat-sen University Cancer Center, Guangzhou, China; ^14^ Department of Pediatrics, Huizhou Central People’s Hospital, Huizhou, China; ^15^ Department of Pediatrics, Zhongshan People’s Hospital, Zhongshan, China; ^16^ Department of Pediatrics, The First Affiliated Hospital of Shantou University Medical College, Shantou, China; ^17^ Department of Pediatrics, Guangzhou First People’s Hospital, Guangzhou, China; ^18^ Department of Pediatrics, Boai Hospital of Zhongshan, Zhongshan, China; ^19^ Department of Pediatrics, Huizhou First People’s Hospital, Huizhou, China

**Keywords:** acute lymphoblastic leukemia, central nervous system leukemia, childhood, prognosis, relapse

## Abstract

**Objectives:**

The prognostic significance of acute lymphoblastic leukemia (ALL) patients with central nervous system leukemia (CNSL) at diagnosis is controversial. We aimed to determine the impact of CNSL at diagnosis on the clinical outcomes of childhood B-cell ALL in the South China Children’s Leukemia Group (SCCLG).

**Methods:**

A total of 1,872 childhood patients were recruited for the study between October 2016 and July 2021. The diagnosis of CNSL depends on primary cytological examination of cerebrospinal fluid, clinical manifestations, and imaging manifestations. Patients with CNSL at diagnosis received two additional courses of intrathecal triple injections during induction.

**Results:**

The frequency of CNLS at the diagnosis of B-cell ALL was 3.6%. Patients with CNSL at diagnosis had a significantly higher mean presenting leukocyte count (*P* = 0.002) and poorer treatment response (*P <*0.05) compared with non-CNSL patients. Moreover, CNSL status was associated with worse 3-year event-free survival (*P* = 0.030) and a higher risk of 3-year cumulative incidence of relapse* *(*P* = 0.008), while no impact was observed on 3-year overall survival (*P* = 0.837). Multivariate analysis revealed that CNSL status at diagnosis was an independent predictor with a higher cumulative incidence of relapse (hazard ratio = 2.809, *P* = 0.016).

**Conclusion:**

CNSL status remains an adverse prognostic factor in childhood B-cell ALL, indicating that additional augmentation of CNS-directed therapy is warranted for patients with CNSL at diagnosis.

## Introduction

Acute lymphoblastic leukemia (ALL) is a heterogeneous disease that accounts for approximately 80% of all leukemias in children. Based on the immunophenotype, ALL cases can be classified as B-cell or T-cell ALL, with B-cell ALL comprising 85%–90% of newly diagnosed cases (1, 2). With recent advances in risk-directed treatment, 5-year event-free survival (EFS) has exceeded 85% and 5-year overall survival (OS) has surpassed 90% for pediatric patients with B-cell ALL in many clinical trials (3, 4). However, central nervous system involvement remains one of the most important challenges in the treatment of childhood B-cell ALL (5).

The prognostic significance of patients with central nervous system leukemia (CNSL) at diagnosis is controversial. A report from the European Organization for Research and Treatment of Cancer (EORTC) Children’s Leukemia Group study 58881 showed that the presence of initial CNS involvement had no prognostic significance for survival (6). In contrast, another report from the EORTC Children’s Leukemia Group study 58951 showed that CNSL at diagnosis remained an independent adverse prognostic factor in children with ALL (7). Additionally, investigators from the Children’s Oncology Group (COG) reported that CNSL at diagnosis predicted inferior outcome and higher rates of CNS relapse in patients with B-cell ALL (8). The resulting differences may be due to different therapeutic protocols.

The South China Children’s Leukemia Group (SCCLG)-ALL-2016 is a prospective, multi-institutional clinical trial involving 18 major hospitals/medical centers (9, 10). We designed the SCCLG-ALL-2016 collaborative protocol based on the backbones of Berlin-Frankfurt-Münster (BFM) ALL-IC-2009 and Guangdong (GD)-2008-ALL (11, 12). In this study, we aimed to determine the impact of CNSL at diagnosis on the clinical outcomes of childhood B-cell ALL in the SCCLG-ALL-2016 trial.

## Materials and methods

### Patients

A total of 1,872 pediatric patients who were diagnosed with B-cell ALL between October 2016 and July 2021 were recruited for the study. The inclusion criteria were: age ≤18 years, treated according to the SCCLG-ALL-2016 protocol. The exclusion criteria were: mature B-ALL, mixed phenotype leukemia, condition secondary to immunodeficiency disease, presence of Down’s syndrome, non-primary, untreated according to the protocol, and incomplete data for the information of CNS status. The study was conducted in accordance with the principles set down in the Declaration of Helsinki and was approved by the Ethics Committee of Sun Yat-sen Memorial Hospital, Sun Yat-sen University. All patients, or the parents/guardians of patients, provided written informed consent. The trial is registered with the Chinese Clinical Trial Registry (https://www.chictr.org.cn/; ChiCTR2000030357).

### CNS status

CNS status was classified into two groups (CNSL and non-CNSL) based on clinical manifestations, imaging (CT/MRI) findings, cerebrospinal fluid (CSF) white blood cells (WBCs), red blood cells (RBCs), and leukemia blasts. Clinical manifestations of CNSL include general neurologic symptoms, signs of cranial nerve palsy, and spinal involvement. CNSL: CSF WBC >5/μl with blasts in non-traumatic lumbar puncture (TLP, >10 erythrocytes/μl), or TLP with a positive Steinherz/Bleyer result (CSF WBC/RBC ≥2×peripheral blood WBC/RBC), or any clinical or imaging evidence of CNSL (13, 14). Otherwise, the other status excluding CNSL was defined as non-CNSL. According to the SCCLG-ALL-2016 protocol, CNSL at diagnosis stratified the patients into the intermediate-risk or high-risk groups. Patients with CNSL at diagnosis received two additional courses of intrathecal triple treatment (methotrexate, dexamethasone, and cytarabine) during induction. During continuation therapy, intrathecal therapy was administered according to risk features. Cranial irradiation was omitted for all patients.

### Treatment response

Early response to treatment was measured as the absolute number of peripheral lymphoblasts at induction therapy. Peripheral blood blasts <1.0 × 10^9^/L on day 8 was considered prednisone good response (PGR), otherwise it was considered prednisone poor response (PPR). Minimal residual disease (MRD) was analyzed at the central protocol laboratory by flow cytometry. MRD positive was defined as ≥0.1% and ≥0.01% on day 15 and day 33, respectively. Complete remission (CR) was defined as less than 5% lymphoblasts in active hematopoietic bone marrow in the absence of clinical evidence of disease at the end of induction. Relapse was defined as the presence of lymphoblasts (≥25%) in the BM or on histological documentation of blasts in extramedullary sites after the achievement of CR.

### Statistical analysis

In cases with small numbers, chi-squared analysis and Fisher’s exact test were used to compare categorical variables. The non-parametric Mann–Whitney *U*-test was applied to continuous variables. EFS was calculated from the time between diagnosis and the first event, including induction failure, relapse, death of any cause, or to the point of last follow-up. OS was measured from the start of initial therapy to death from any cause or to the point of last follow-up. The cumulative incidence of relapse (CIR) was defined as the time from the achievement of CR until the occurrence of the first relapse. The Kaplan–Meier model was applied to calculate 3-year EFS and OS, which were compared using the log-rank test. The three-year CIR was estimated by the Fine–Gray subdistribution hazard model, and differences were analyzed by Gray’s test. The competing event for relapse was death during remission. Prognostic factors were examined by multivariate Cox regression analysis. *P*-values of <0.05 were considered statistically significant (two-tailed testing). The data were analyzed with the Statistical Package for the Social Sciences (SPSS^®^) version 24.0 (IBM Corporation, Armonk, NY, USA).

## Results

### Patients’ characteristics

Overall, among 1,872 patients with childhood B-cell ALL, 68 (3.6%) demonstrated CNSL status at diagnosis. Neurological symptoms such as headaches, vomiting, and convulsions were observed in eight patients among the patients with CNSL. However, the clinical features of CNSL were not associated with imaging or cytology positivity in our study. As shown in **Table 1**, there were no differences in gender and age distribution between CNSL and non-CNSL patients. Nevertheless, compared with non-CNSL, childhood B-cell ALL with CNSL at diagnosis had a significantly higher median leukocyte count (22.8 × 10^9^/L vs. 9.0 × 10^9^/L, *P* = 0.002). Among all the patients with CNSL at diagnosis, 24 cases (35.3%) and 44 cases (64.7%) were stratified into the intermediate-risk and high-risk groups, respectively. Additionally, no significant differences were found in the frequencies of fusion genes including *BCR/ABL*, *MLL-r*, *ETV6/RUNX1*, and *E2A/PBX1* between patients with CNSL and non-CNSL. In total, 27 (1.4%) patients received hematopoietic stem cell transplantation in our study. However, the percentage of hematopoietic stem cell transplantation was equally distributed between the CNSL group and the non-CNSL group (*P* = 0.257).

**Table 1:** Characteristics of childhood B-ALL with or without CNSL at diagnosis.

Characteristics	Total	CNS status		*P*-value
	1,872	Non-CNSL (n = 1,804)	CNSL (n = 68)	
Gender, n (%)				0.798
Male	1,073 (57.3%)	1,033 (57.3%)	40 (58.8%)	
Female	799 (42.7%)	771 (42.7%)	28 (41.2%)
Age (y), median (range)				0.652
Age (y), n (%)	4.4 (0.5–17.4)	4.4 (0.5–17.4)	4.9 (0.6–14.1)	0.317
<10	1,618 (86.4%)	1,562 (86.6%)	56 (82.4%)	
≥10	254 (13.6%)	242 (13.4%)	12 (17.6%)
WBC (×10^9^/L), median (range)				0.002
WBC (×10^9^/L), n (%)	9.3 (0.16–1095)	9.0 (0.16–1095)	22.8 (0.93–618.7)	0.001
<50	1,540 (82.3%)	1,494 (82.8%)	46 (67.6%)	
≥50	332 (17.7%)	310 (17.2%)	22 (32.4%)
Risk group, n (%)				<0.001
Low risk	454 (24.3%)	454 (25.2%)	0 (0%)	
Intermediate risk	994 (53.1%)	950 (52.7%)	44 (64.7%)
High risk	424 (22.6%)	400 (22.2%)	24 (35.3%)
*BCR/ABL* status, n (%)				0.170
Negative	1,750 (94.5%)	1,690 (94.7%)	60 (90.9%)	
Positive	101 (5.5%)	95 (5.3%)	6 (9.1%)
*MLL-r* status, n (%)				0.178
Negative	1,810 (97.8%)	1,747 (97.9%)	63 (95.5%)	
Positive	41 (2.2%)	38 (2.1%)	3 (4.5%)
*ETV6/RUNX1* status, n (%)				0.261
Negative	1,596 (86.2%)	1,536 (86.1%)	60 (90.9%)	0.767
Positive	255 (13.8%)	249 (13.9%)	6 (9.1%)
*E2A/PBX1* status, n (%)			
Negative	1,769 (95.6%)	1,705 (95.5%)	64 (97.0%)	0.257
Positive	82 (4.4%)	80 (4.6%)	2 (3.0%)
HSCT, n (%)			
No	1,845 (98.6%)	1,779 (98.6%)	66 (97.1%)	
Yes	27 (1.4%)	25 (1.3%)	2 (2.9%)
Prednisone response, n (%)				0.005
Good	1,721 (92.4%)	1,665 (92.8%)	56 (83.6%)	
Poor	141 (7.6%)	130 (7.2%)	11 (16.4%)
D15 MRD, n (%)				0.033
<0.1%	481 (25.8%)	471 (26.2%)	10 (14.7%)	
≥0.1%	1,382 (74.2%)	1,324 (73.8%)	58 (85.3%)
D33 MRD, n (%)				0.044
<0.01%	475 (25.8%)	465 (26.2%)	10 (15.2%)	
≥0.01%	1,365 (74.2%)	1,309 (73.8%)	56 (84.8%)

ALL, acute lymphoblastic leukemia; CNS, central nervous system; CNSL, central nervous system leukemia; EFS, event-free survival; HSCT, hematopoietic stem cell transplantation; MRD, minimal residual disease; WBC, white blood cell.

### Clinical outcomes

Treatment response was measured by the prednisone response on day 8, as well as MRD on both days 15 and 33. The rate of PGR for patients with CNSL and non-CNSL was 83.6% and 92.8%, respectively, and the difference was statistically significant (*P* = 0.005). Moreover, CNSL at diagnosis was associated with a higher rate of MRD positive on day 15 (85.3% vs. 73.8%, *P* = 0.033) and day 33 (84.8% vs. 73.8%, *P* = 0.044), respectively.

**Figure 1:**
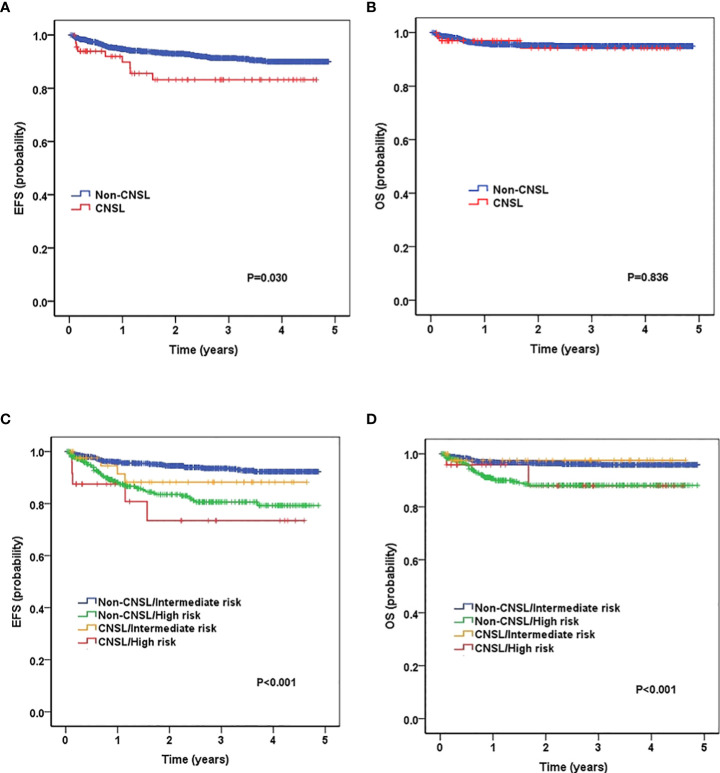
Survival curves of childhood B-cell ALL patients according to central nervous system (CNS) status, and according to the combined CNS status and risk stratification. **(A)** Probability of EFS (event-free survival) for patients with CNSL and non-CNSL at diagnosis. **(B)** Probability of overall survival (OS) for patients with CNSL and non-CNSL at diagnosis. **(C)** Probability of EFS for patients according to the combined CNS status and risk stratification. **(D)** Probability of OS for patients according to the combined CNS status and risk stratification.

We then evaluated the survival data for all childhood patients. As shown in **Figures 1A, B**, patients with CNSL at diagnosis had a significantly worse 3-year EFS (83.2 ± 5.2%) than non-CNSL patients (91.3 ± 0.8%, *P* = 0.030). However, we found that CNSL at diagnosis yielded no significant differences in 3-year OS (94.3 ± 3.4% vs. 95.1 ± 0.6%, *P* = 0.836) between these two groups. We further evaluated the prognostic impact of CNSL and the risk group in patients by stratification analysis. In terms of 3-year EFS (**Figure 1C**), CNSL had an adverse impact trend on the survival of patients restricted to the intermediate-risk group (88.2 ± 5.6% vs. 93.5 ± 0.9%, *P* = 0.256) or the high-risk group (73.4 ± 10.8% vs. 80.6 ± 2.5%, *P* = 0.345), but the difference was not statistically significant. In terms of 3-year OS (**Figure 1C**), we found that CNSL had no significant impact in the survival of patients restricted to the intermediate-risk group (*P* = 0.751) or the high-risk group (*P* = 0.905).

### CIR and distribution of relapses

**Figure 2:**
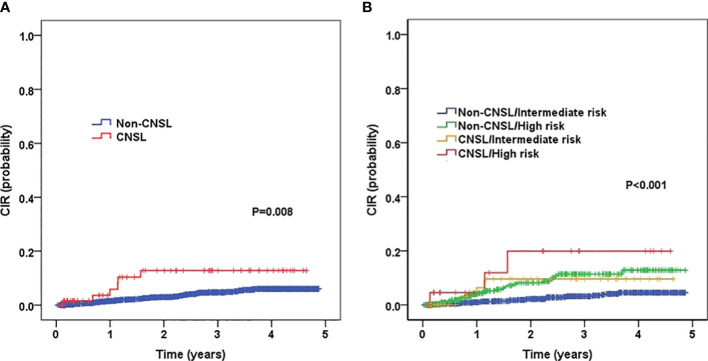
Probability of CIR (cumulative incidence rates) for childhood B-cell ALL patients according to central nervous system (CNS) status, and according to the combined CNS status and risk stratification. **(A)** Probability of CIR for patients with CNSL and non-CNSL at diagnosis. **(B)** Probability of CIR for patients according to the combined CNS status and risk stratification.

The 3-year CIR for patients with CNSL and non-CNSL was 12.8 ± 4.9% and 4.7 ± 0.6%, respectively, and the difference was statistically significant (*P* = 0.008) (**Figure 2A**). By stratification analysis (**Figure 2B**), we found that patients with CNSL at diagnosis had a high-risk trend of 3-year CIR in the intermediate-risk group (9.6 ± 5.3% vs. 3.2 ± 0.7%, *P* = 0.060). However, when restricted to the high-risk group, no significant difference was found between CNSL and non-CNSL patients in terms of 3-year CIR (19.9 ± 10.6% vs. 11.4 ± 2.2%, *P* = 0.259).

We further investigated the distribution of relapses according to CNS status at diagnosis. As shown in **Table 2**, patients with CNSL at diagnosis had a higher risk of relapse (8.8% vs. 3.5%, *P* = 0.036). Notably, CNSL at diagnosis was associated with a higher risk of relapse with CNS involvement (4.4% vs. 0.6%, *P* = 0.010) and isolated CNS relapse (2.9% vs. 0.4%, *P* = 0.040), respectively.

**Table 2:** Distribution of relapse according CNS status at diagnosis.

Relapse	Total (n = 1,872)		Non-CNSL (n = 1,804)		CNSL (n = 68)		*P-*value
	No. of patients	% of relapses	No. of patients	% of relapses	No. of patients	% of relapses	
All relapses	69	3.7	63	3.5	6	8.8	0.036
Relapse with CNS involvement	13	0.7	10	0.6	3	4.4	0.010
Isolated CNS relapse	9	0.5	7	0.4	2	2.9	0.040
BM plus CNS relapse	4	0.2	3	0.2	1	1.5	0.138
Isolated BM relapse	52	2.8	49	2.7	3	4.4	0.434
Other relapses (e.g. testis)	4	0.2	4	0.2	0	0	1.000

CNS, central nervous system; CNSL, central nervous system leukemia; BM, bone marrow.

### Multivariate analyses

**Table 3** summarizes the results of multivariate analyses for the complete cohort of patients with childhood ALL. In the multivariable survival analysis for EFS, OS, and CIR, we included CNS status with other risk factors in the Cox model, including sex, age, WBC, prednisone response on day 8, and MRD on both days 15 and 33 as covariates. Our results showed that CNSL at diagnosis did not reach significance either for EFS (*P* = 0.217) or OS (*P* = 0.323). However, we identified that CNSL status was an independent predictor with a higher CIR (hazard ratio = 2.809, *P* = 0.016). In this model, age (≥10-year-old) and WBC (≥50 × 10^9^/L) were associated with worse EFS, while age (≥10-year-old) and sex (male) were associated with higher CIR.

**Table 3:** Multivariate analysis for EFS, OS and CIR in childhood B-ALL.

Outcome	Variable	Hazard ratio (95% CI)	*P*-value
EFS	CNSL	1.622 (0.752–3.498)	0.217
	Male	1.191 (0.824–1.721)	0.352
	Age ≥10 y	1.931 (1.253–2.974)	0.003
	WBC ≥5 × 10^9^/L	1.580 (1.027–2.429)	0.037
	D8 PPR	1.547 (0.868–2.755)	0.139
	D15 MRD ≥0.1%	1.391 (0.292–6.615)	0.679
	D33 MRD ≥0.01%	1.136 (0.242–5.339)	0.872
OS	CNSL	0.368 (0.051–2.668)	0.323
	Male	0.925 (0.564–1.516)	0.757
	Age ≥10 y	1.769 (0.984–3.179)	0.056
	WBC ≥50 × 10^9^/L	1.394 (0.777–2.501)	0.266
	D8 PPR	2.015 (0.978–4.148)	0.057
	D15 MRD ≥0.1%	1.788 (0.152–20.991)	0.644
	D33 MRD ≥0.01%	1.659 (0.143–19.244)	0.686
CIR	CNSL	2.809 (1.209–6.526)	0.016
	Male	1.809 (1.073–3.049)	0.026
	Age ≥ 10 y	1.927 (1.070–3.471)	0.029
	WBC ≥ 50×10^9^/L	1.766 (0.994–3.138)	0.052
	D8 PPR	1.069 (0.444–2.572)	0.882
	D15 MRD ≥ 0.1%	1.223 (0.183–8.182)	0.836
	D33 MRD ≥ 0.01%	0.951 (0.145–6.235)	0.958

CI, confidence interval; CIR, cumulative incidence rates; CNSL, central nervous system leukemia; EFS, event-free survival; MRD, minimal residual disease; OS, overall survival; PPR, prednisone poor response.

## Discussion

CNSL is caused by the infiltration of leukemia cells into the CNS. The diagnosis of CNSL was made by primary cytological examination of the CSF, clinical manifestations, and imaging manifestations. In our SCCLG cohort, the frequency of CNLS at diagnosis among 1,872 patients with childhood B-cell ALL was 3.6%. This result was higher than that of other childhood B-cell ALL reports (range 1.3%–1.7%) (6, 7). This difference may be explained in part by the different classification of CNS status and various study populations. Moreover, we found that childhood B-cell ALL with CNSL at diagnosis had a significantly higher median leukocyte count. However, in our study, no association was found between the CNS status and the frequency of fusion genes, including *BCR/ABL*, *MLL-r*, *ETV6/RUNX1*, and *E2A/PBX1*. Currently, hyperleukocytosis upon diagnosis is considered a risk factor for CNS involvement in B-cell ALL (15). Nevertheless, some reports have shown that *BCR/ABL*, *MLL-r*, and *E2A/PBX1* are associated with a higher incidence of CNSL in B-cell ALL (16–18).

There has been controversy about the significance of CNS involvement in childhood ALL. A report from the Nordic Society of Paediatric Haematology and Oncology (NOPHO) showed that the post induction bone marrow MRD did not differ between patients with CNS involvement and those without CNS involvement. Moreover, the 12-year EFS for patients with CNSL at diagnosis did not differ from that in patients with non-CNSL (19). Another report from the Dutch Childhood Oncology Group also showed no significant difference in 10-year EFS between patients with CNSL and non-CNSL at diagnosis (20). These findings were in accordance with the results of the EORTC Children’s Leukemia Group study 58881 (6). However, a study from the ALL-BFM-95 trial showed that patients with CNSL at diagnosis had the worst prognosis, with a 5-year EFS estimate of 50%, and these patients had a higher risk of CNS relapses (21). Another trial from the EORTC Children’s Leukemia Group study 58951 showed that CNSL at diagnosis was associated with poorer induction response, shorter 5-year EFS and OS, and a higher risk of relapse (7). A large cohort of 8,379 patients with B-cell ALL from the COG confirmed that patients with CNSL at diagnosis predicted higher MRD post induction and higher rates of CNS relapse (8). In our cohort of 1,872 childhood B-cell ALL patients, we found that CNSL at diagnosis had a poorer treatment response and worse EFS than non-CNSL patients. Moreover, CNSL at diagnosis was associated with a higher risk of CNS relapse and was an adverse independent factor for CIR in multivariate analysis. The resulting differences may be due to the differences in the efficacy of systemic and CNS-directed therapies among the study groups.

The optimal management of CNS disease in patients with ALL remains uncertain (5, 22). A meta-analysis of more than 16,000 patients recruited from 10 cooperative study groups demonstrated that cranial radiation as first-line therapy did not impact the risk of relapse in children with ALL (23). Recently, the use of cranial radiation has become contentious because of its late adverse effects, especially in children, such as neurocognitive dysfunction, growth impairment, precocious puberty, and an enhanced risk of developing brain tumors (24–26). Intrathecal chemotherapy is considered an effective management strategy by direct injection into CSF with maximized drug exposure in the CNS. A trial in the St. Jude Total Therapy Study showed that additional intrathecal therapy during early induction contributed to improved CNS control without excessive toxicity in high-risk patients (27). However, it has been reported that intrathecal chemotherapy is much more likely to cause catastrophic consequences (28–30). Recently, Qi et al. (31) found that CD19-specific chimeric antigen receptor T-cell-based therapy might provide a potential treatment option for B-cell ALL patients with CNSL. Alternately, Tang et al. reported results from the CCCGALL-2015 trial, where they found that upfront dexamethasone might reduce leukemic blasts in the blood and CNS before diagnostic lumbar puncture and general anesthesia might reduce the risk of TLP, while CSF flow cytometry might allow a more accurate diagnosis (32). Other studies targeting the components of the CNS niche and promoting the introduction of chemotherapy drugs into the CNS are worthy of further investigation (33–35).

There were some limitations to our study. First, the time period of follow-up in our study was short. We only analyzed the 3-year survival between patients with CNSL and non-CNSL at diagnosis. A report from the EORTC Children’s Leukemia Group study 58881 has shown that the prognosis was worse for patients with CNSL in terms of 3-year survival, while it was not at long-term follow-up (6). Thus, a long-term follow-up study is needed to further address the prognostic significance of CNSL at diagnosis. Second, we did not classify CNSL at diagnosis into different groups, such as abnormal cytological examination of CSF or imaging manifestations. However, Levinsen et al. demonstrated that the long-term EFS for patients with leukemia mass on neuroimaging did not differ from CNSL patients with negative scans (19). Third, our study was a lack of data regarding the immunophenotype of CNS status. Recently, it has been demonstrated that flowcytometric evaluation of CSF in childhood ALL better identifies CNS involvement than conventional cytomorphology. Further studies with multiparametric flow cytometry are warranted to obtain higher quality evidence.

In summary, we analyzed the prognostic impact of CNSL for the diagnosis of childhood B-cell ALL in the SCCLG trial. Our findings showed that patients with CNSL at diagnosis had an independent adverse prognostic factor for CNS relapse. Additional augmentation of CNS-directed therapy is warranted for managing CNS involvement.

## Data availability statement

The original contributions presented in the study are included in the article/supplementary material. Further inquiries can be directed to the corresponding author.

## Ethics statement

The studies involving human participants were reviewed and approved by the Ethics Committee of Sun Yat-sen Memorial Hospital, Sun Yat-sen University. Written informed consent to participate in this study was provided by the participants’ legal guardian/next of kin.

## Author contributions

L-HX and YL participated in project design, analysis, interpretation, and manuscript drafting. XG, NL, L-HY, H-RM, W-QW, L-BH, and M-CZ obtained and assembled data. CT, H-QC, Q-WC, X-JL, Z-JZ, R-YL, Q-RL, B-YW, L-NW, X-LK, G-HC, and J-PF analyzed and interpreted the data. All authors contributed to the article and approved the submitted version.

## Funding

This work was supported by the Guangdong Basic and Applied Basic Research Foundation (2020A1515010312) and the New Sunshine Charity Foundation.

## Acknowledgments

We thank all members of our partner hospitals for their efforts in collecting information and all patients who have agreed to supply their data.

## Conflict of interest

The authors declare that the research was conducted in the absence of any commercial or financial relationships that could be construed as a potential conflict of interest.

## Publisher’s note

All claims expressed in this article are solely those of the authors and do not necessarily represent those of their affiliated organizations, or those of the publisher, the editors and the reviewers. Any product that may be evaluated in this article, or claim that may be made by its manufacturer, is not guaranteed or endorsed by the publisher.
